# Does the gene matter? Genotype–phenotype and genotype–outcome associations in congenital melanocytic naevi[Link]

**DOI:** 10.1111/bjd.18106

**Published:** 2019-08-09

**Authors:** S. Polubothu, N. McGuire, L. Al‐Olabi, W. Baird, N. Bulstrode, J. Chalker, D. Josifova, D. Lomas, J. O'Hara, J. Ong, D. Rampling, P. Stadnik, A. Thomas, E. Wedgeworth, N.J. Sebire, V.A. Kinsler

**Affiliations:** ^1^ Genetics and Genomic Medicine University College London Great Ormond Street Institute of Child Health London U.K.; ^2^ Paediatric Dermatology Great Ormond Street Hospital for Children NHS Foundation Trust London U.K.; ^3^ Paediatric Plastic Surgery Great Ormond Street Hospital for Children NHS Foundation Trust London U.K.; ^4^ Paediatric Malignancy Unit Great Ormond Street Hospital for Children NHS Foundation Trust London U.K.; ^5^ Clinical Genetics Guy's and St Thomas’ Hospital NHS Foundation Trust U.K.; ^6^ Paediatric Pathology Great Ormond Street Hospital for Children NHS Foundation Trust London U.K.; ^7^ Department of Dermatology Guy's and St Thomas’ Hospital NHS Foundation Trust U.K.

## Abstract

**Background:**

Genotype–phenotype studies can identify subgroups of patients with specific clinical features or differing outcomes, which can help shape management.

**Objectives:**

To characterize the frequency of different causative genotypes in congenital melanocytic naevi (CMN), and to investigate genotype–phenotype and genotype–outcome associations.

**Methods:**

We conducted a large cohort study in which we undertook *MC1R* genotyping from blood, and high‐sensitivity genotyping of *NRAS* and *BRAF* hotspots in 156 naevus biopsies from 134 patients with CMN [male 40%; multiple CMN 76%; projected adult size (PAS) > 20 cm, 59%].

**Results:**

Mosaic *NRAS* mutations were detected in 68%, mutually exclusive with *BRAF* mutations in 7%, with double wild‐type in 25%. Two separate naevi were sequenced in five of seven patients with *BRAF* mutations, confirming clonality. Five of seven patients with *BRAF* mutations had a dramatic multinodular phenotype, with characteristic histology distinct from classical proliferative nodules. *NRAS* mutation was the commonest in all sizes of CMN, but was particularly common in naevi with PAS > 60 cm, implying more tolerance to that mutation early in embryogenesis. Facial features were less common in double wild‐type patients. Importantly, the incidence of congenital neurological disease, and apparently of melanoma, was not altered by genotype; no cases of melanoma were seen in *BRAF*‐mutant multiple CMN, however, this genotype is rare.

**Conclusions:**

CMN of all sizes are most commonly caused by mutations in *NRAS*. *BRAF* is confirmed as a much rarer cause of multiple CMN, and appears to be commonly associated with a multinodular phenotype. Genotype in this cohort was not associated with differences in incidence of neurological disease in childhood. However, genotyping should be undertaken in suspected melanoma, for guidance of treatment.

**What's already known about this topic?**

- Multiple congenital melanocytic naevi (CMN) have been shown to be caused by *NRAS* mosaic mutations in 70–80% of cases, by *BRAF* mosaicism in one case report and by inference in some previous cases.
- There has been debate about genotypic association with different sizes of CMN, and no data on genotype–outcome.

**What does this study add?**

- *NRAS* mosaicism was found in 68%, *BRAF* in 7% and double wild‐type in 25% of cases of CMN.
- *NRAS* was the commonest mutation in all sizes of CMN, but was nearly universal in projected adult size > 60 cm.
- *BRAF* is often associated with a distinct multinodular clinical/histological phenotype.
- Adverse outcomes did not differ between genotypes on current numbers.

## Abstract 2

https://doi.org/10.1111/bjd.18747 available online

The diagnosis of congenital melanocytic naevi (CMN) covers a wide phenotypic spectrum. Small single CMN are very common birthmarks, present in approximately 1% of all neonates,^1^, ^2^, ^3^, ^4^, ^5^, ^6^ are not associated with extracutaneous abnormalities, and have a very low risk of transformation to melanoma.^7^, ^8^, ^9^, ^10^ At the other end of the spectrum, multiple CMN (defined as two or more melanocytic naevi at birth) can cover up to 80% of the body surface area, can be associated with serious extracutaneous abnormalities, such as congenital neurological disease,^11^, ^12^, ^13^, ^14^ typical facial features,^15^ underdevelopment of fat and muscle in naevoid areas, and endocrinological disturbance.^16^ The risk of melanoma in childhood varies with the congenital phenotype (both cutaneous and neurological), between 1% and 12% in prospective studies.^13^, ^17^ Where CMN are associated with extracutaneous abnormalities, the term CMN syndrome^15^ is preferred to the historical term neurocutaneous melanosis, as the latter does not encompass the whole range of neurological associations, and critically does not distinguish between benign congenital neurological disease, and acquired central nervous system melanoma.

The molecular basis of multiple CMN and CMN syndrome was only recently clearly established. As this is a sporadic condition and had been described in one twin of monozygotic pairs,^18^, ^19^ it was proposed 25 years ago to be due to a postzygotic mutation *in utero*, and therefore to represent a mosaic disorder.^20^ However, reports of occasional familial cases^21^, ^22^, ^23^ later led to the hypothesis that multiple CMN were due to paradominant inheritance with allelic loss,^24^ and later still to a patchy manifestation of a polygenic trait.^25^ In the interim, many variants were described in single samples of CMN from cohorts of patients, including *TP53,* 26 *NRAS,* 26, 27, 28, 29, 30 *BRAF,* 31, 32, 33, 34, 35 *GNAQ* 29 and *MC1R*.^26^, ^36^ A systematic study established that the same mutation was present in multiple affected tissues from one individual, and absent from unaffected tissues including blood,^37^ which is the cornerstone of demonstrating clonality and, therefore, by inference, likely causal mutations in mosaicism where there are multiple lesions. This study demonstrated *NRAS* mutations affecting codon 61 [p.(Q61K) or (p.Q61R)] in 12 of 15 patients. Subsequent studies of a rarer phenotype, multiple naevus spilus‐type CMN, revealed different changes in *NRAS* [p.(Q61H), p.(G13R), p.(Q61L)].^38^, ^39^ Since then, further publications of single samples from patients with CMN have confirmed the predominance of *NRAS* mutations in large or giant CMN.^40^, ^41^

The role of *BRAF* in multiple CMN has been even more recently clarified. Previous studies had demonstrated *BRAF* p.(V600E) mutations in single samples of CMN,^31^, ^32^, ^33^, ^34^, ^35^, ^40^ with two reported cases of *BRAF* activation secondary to a chromosomal translocation involving 7q, demonstrated in melanocytes derived from the main CMN.^35^ Furthermore, multiple CMN is an established feature in patients with ring chromosome 7, with evidence of somatic mosaicism leading to significant gain of chromosome 7 within the CMN.^42^, ^43^ However, a role for *BRAF* in CMN was debated, with no evidence for *BRAF* variants in true congenital naevi in one study.^27^ A single case of multiple CMN where mutant *BRAF* was detected in more than one naevus from the same patient has now been published,^44^ confirming clonality in that patient as opposed to a second hit, and effectively confirming the pathogenesis in previous reports of multiple CMN or CMN syndrome. Single cases of fusion transcripts as potential conduits of multiple CMN have also been recently described, one each of *ZEB2‐ALK* and *SOX5‐RAF1*.^45^

Genotype has already been demonstrated to be clinically relevant in terms of management, with *NRAS*‐mutant melanoma treated with MEK inhibition leading to symptomatic improvement, though not cure.^46^ We sought to explore genotype–phenotype and genotype–outcome associations in a large cohort of patients, to improve our understanding of the effect of genotype on clinical outcome, and to assess whether routine genotyping is relevant in routine clinical practice.

## Patients and methods

### Genotypic and phenotypic characterization of patients

Children with CMN seen in the paediatric dermatology department of a tertiary referral centre between January 2015 and January 2018 were offered participation in a genotyping study, approved by the local NHS Research Ethics Committee (London Bloomsbury). This involved a blood sample for *MC1R* genotyping, and/or a single 4‐mm punch skin biopsy for genotyping for *NRAS* and *BRAF* mutational ‘hotspots’, and/or genotyping from archival formalin‐fixed paraffin‐embedded (FFPE) tissue.

DNA was extracted by standard methods, and directly without skin culture from fresh skin samples (DNeasy Blood & Tissue Kit; Qiagen, Hilden, Germany), or from FFPE CMN tissue (Maxwell^®^ RSC DNA FFPE Kit, Promega Corporation, Madison, WI, U.S.A.). All patients underwent deep clinical phenotyping, including assessment of facial features as previously published.^15^ Phenotyping was by two different classifications, an in‐house classification as previously published,^13^, ^17^, ^37^ and a recent consensus classification from the literature.^47^

DNA was obtained from peripheral blood samples in 75 individuals, and from 156 samples of CMN in 134 individuals. Skin DNA was initially genotyped in the hospital diagnostic laboratory for hotspot mutations in *NRAS* leading to amino acid changes in codon 61, and in *BRAF* affecting codon 600. Samples that were negative for both of these hotspots (‘double wild‐type’) were additionally sequenced for *NRAS* mutations affecting codons 12 and 13. Genotyping for *NRAS* was by two methods, namely touchdown polymerase chain reaction (PCR) and Sanger sequencing (sensitivity down to 10% mutant allele detection), and High Resolution Melt (HRM) (sensitivity down to 1%). Genotyping for *BRAF* mutations was by three diagnostic‐grade methods initially, as at the time *BRAF* mutation was not an established cause of multiple CMN: Sanger sequencing, HRM, and allele‐specific real‐time PCR (*BRAF* Codon 600 Mutational Analysis Kit, EntroGen, Inc., Woodland Hills, CA, U.S.A.). The latter was later discontinued, as the concordance between HRM and real‐time PCR was 100%.Where a *BRAF* p.(V600E) mutation was found, a second biopsy from a separate skin lesion was offered to the patient, as well as a blood sample, and both were tested by the same methods. *MC1R* genotyping was performed in all patients by Sanger sequencing of lymphocyte DNA extracted from whole blood, as previously described.^36^ Genotyping results for *NRAS, BRAF* and *MC1R* were ultimately available for 117, 96 and 75 patients, respectively, as in some cases results were not possible from FFPE tissue, or patients did not wish to have either a blood test or biopsy.

### Statistical analysis

Genotyping data were included as independent variables in multiple logistic regression modelling of clinical phenotype variables using IBM SPSS Statistics 24·0 (IBM, Armonk, NY, U.S.A.). Specifically, the associations modelled were between *NRAS* and *MC1R* genotype (mutant or wild‐type) and projected adult size (PAS) of the largest CMN, typical facies and congenital neurological disease on magnetic resonance imaging (MRI). A statistical correction was applied for multiple testing, which reduced the *P*‐value for significance to *P* < 0·0125. *BRAF* genotype was not used to model phenotype statistically as the total number of patients was too low, and similarly melanoma as an outcome was not modelled separately.

### Cell culture and cell treatment

Naevus cells were isolated and cultured from fresh CMN tissue. Briefly, tissue was incubated in trypsin 0·25% for 4 h at 37 °C to allow removal of the epidermis. Following this, the reticular dermis was dissected out and digested in dispase/collagenase for a further 6 h, before neutralizing the digest with complete melanocyte media [Ham's F‐10 Nutrient Mix (11550043; Thermo Fisher Scientific, Waltham, MA, U.S.A.) supplemented with Ultroser G, fetal bovine serum, tissue plasminogen activator, cholera toxin, human stem cell factor, 3‐isobutyl‐1‐methylxanthine, fibroblast growth factor, endothelin, penicillin/streptomycin and amphotericin B]. A single‐cell suspension was achieved by filtering cells through a 70‐μm followed by a 40‐μm cell strainer before seeding cells in complete media into tissue culture flasks. When flasks reached 70% confluence, DNA was extracted directly from pelleted cells washed once with phosphate‐buffered saline using DNeasy Tissue Kit (Qiagen). Sanger sequencing was performed as described above. BRAF V600E naevus cells were additionally seeded onto laminin‐coated slides, fixed with 4% paraformaldehyde and permeabilized with 0·01% Triton X‐100, before blocking for 1 h at room temperature in 10% bovine serum albumin. Cells were incubated overnight at 4 °C with BRAF V600E antibody 1 : 1000 (ab200535; Abcam, Cambridge, U.K.), with appropriate negative controls, followed by incubation with goat anti‐rabbit IgG‐conjugated Alexa Fluor 488 secondary antibody (A‐11034; Thermo Fisher Scientific) for 1 h at room temperature. Slides were washed and mounted, and imaged using a Zeiss Axio Observer four‐colour fluorescence microscope (Carl Zeiss Microscopy GmbH, Jena, Germany).

## Results

### Demographic and phenotypic characterization of the cohort

Details of the demographic and phenotypic characterization are shown in Table 1. This cohort is at the more serious phenotypic end of the spectrum of children with CMN, with 76% having multiple CMN (two or more at birth), 59% and 32% with PAS of the largest naevus > 20 cm and > 60 cm, respectively, and 29% with congenital neurological involvement on screening MRI; 69% had at least three typical facial features of CMN syndrome. Eight cases with melanoma were included, seven of whom were in our recent melanoma cohort review.^17^

**Table 1:** Summary of genotype and phenotype of cohort

Sex	MRI	Neurological and/or developmental problems	*NRAS* genotype	*BRAF* genotype	*MC1R* genotype	Multiple CMN	Postnatal/ recurrent nodules	Classic facies
Male 53 (39·6)	Normal 67 (71·3)	Yes 23 (18·3)	Mutation positive 80 (68·4) p.(Q61K) 59 (73·8) p.(Q61R) 17 (21·3) p.(Q61H) 3 (3·8) p.(G13R) 1 (1·25)	*BRAF* p.(V600E) 7 (7·3)	At least one *MC1R* variant 47 (62·7)	Multiple 94 (75·8)	Present 26 (22·4)	Present 66 (68·8)
Female 81 (60·4)	Abnormal 27 (28·7)	None 103 (81·7)	Mutation negative 37 (31·6)	Mutation negative 89 (92·7)	No *MC1R* variants 28 (37·3)	Single 30 (24·2)	Absent 89 (76·7)	Absent 30 (31·3)
Missing *n* = 0	Missing *n* = 40	Missing *n* = 8	Missing *n* = 17	Missing *n* = 38	Missing *n* = 59	Missing *n* = 10	Missing *n* = 19	Missing *n* = 38

All data are presented as *n* (%) unless otherwise stated. MRI, magnetic resonance imaging; CMN, congenital melanocytic naevi.

### Genotypic characterization of the cohort

Genotyping results are shown in Table 1 *. NRAS* variants were detected in 68%, all but one affecting codon 61. The mutational profile revealed a predominance of p.(Q61K) (74% of the 68%) over p.(Q61R) (21% of the 68%), consistent with previous smaller studies,^28^, ^37^ but differing from the mutational profile for non‐CMN‐associated melanoma, where these two variants have equal frequency.^48^, ^49^ For p.(Q61K) this was usually due to c.(C181A); however, two patients carried small indels, c.180_181delinsTA and c.181_183delinsAAG, the former not previously described in CMN but described once in melanoma,^50^ and the latter described once before in three cases of CMN^40^ and once in melanoma.^51^ Additionally, three patients had *NRAS* p.(Q61H) mutations, all of which were in naevus spilus‐type CMN, two of whom were previously published in a naevus spilus‐type CMN study.^38^ Similarly, the one patient with a mutation affecting a different codon [p.(G13R)] was previously published with a naevus spilus‐type CMN phenotype.^38^ Eleven patients with mutant *NRAS*, and two patients who were double wild‐type, were previously published in our original CMN genotype study.^37^

*BRAF* c.1799T>A*,* p.(V600E) was detected in naevi from 7% (*n* = 7) of the cohort, and was also found in a second biopsy of a separate CMN in all patients where it was possible to obtain this tissue (five of seven).

In the remaining 25% of patients, both *NRAS* and *BRAF* were not mutated at these hotspots (double wild‐type), and also did not carry variants affecting *NRAS* codons 12 or 13. This is again consistent with previous studies of CMN but inconsistent with the mutational profile of *NRAS*‐mutant melanoma where codons 12/13 contribute 8% to the total *NRAS* variants.^49^

### Genotype–phenotype analysis

**Figure 1:**
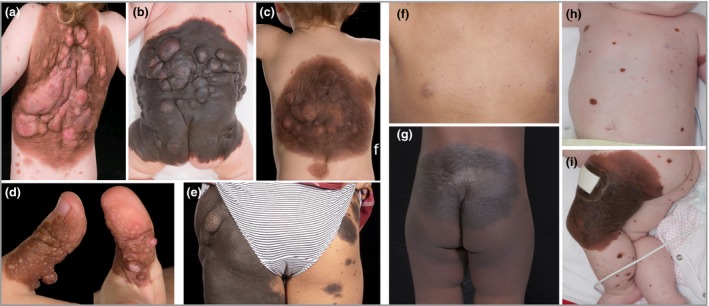
(a–e) Cutaneous phenotype of the five *BRAF*‐mosaic patients with a multinodular phenotype. (f–i) Cutaneous phenotype of two *BRAF*‐mosaic patients without the multinodular phenotype, both presenting with a medium congenital melanocytic naevus and > 200 smaller naevi [patient 6 (f, g) from Table 2, and patient 7 (h, i) from Table 2].

In five of seven *BRAF*‐mutant patients there was a striking multinodular clinical phenotype within the largest CMN (Fig. 1, detailed clinical phenotype in Table 2), with densely packed, uniform multiple benign nodules appearing both pre‐ and postnatally. This is highly suggestive that this rare genotype is linked to this rare multinodular phenotype. From what we have observed, this phenotype can be distinguished from other types of multinodularity by the uniformity of the size and appearance of the nodules within a patient, and the frequent presence of a mobile, firm‐to‐hard lump within a softer exterior to each of the nodules. Multiple similar uniform pre‐ and postnatal nodules were also seen in 4% of double wild‐type patients, but not in *NRAS*‐mutant patients.

**Table 2:** Detailed phenotype of *BRAF*‐mutant multiple congenital melanocytic naevus (CMN)

Patient	Sex	Age (years)	Neuro‐development	Krengel classification^47^	Classic facies	Single or multiple	MRI	Skin samples tested, *n*	Comorbidities
1	Female	3	Speech delay	CMN PAS L2 > 30–40 cm CMN localization Trunk: upper back, middle back Satellites S3 C1 R0 N2 H2	Yes	Multiple	Normal	2	Nil
2	Male	8	Normal	CMN PAS G1 > 40–60 cm CMN localization Trunk: middle back, lower back, abdomen, flank, gluteal region, genital region Satellites S2 C0 R0 N2 H0	Yes	Multiple	Not performed	2	Nil
3	Female	0·5	Normal	CMN PAS L2 > 30–40 cm CMN localization Trunk: upper back, middle back Satellites S2 C0 R0 N2 H1	N/A	Multiple	Intraparenchymal melanosis	2	Nil
4	Female		Normal	CMN PAS G2 > 60 cm CMN localization Trunk: gluteal region; extremities: thigh; lower leg Satellites S3 C0 R1 N2 H2	No	Multiple	Not performed	1	Nil
5	Female		Normal	CMN PAS M1 1·5–10 cm CMN localization Extremities: hand Satellites S1 C1 R0 N2 H0	Missing	Multiple	Not performed	1	Nil
6	Male	4	Speech delay	CMN PAS L1 > 20–30 cm CMN localization Trunk: lower back, gluteal region Satellites S3 C0 R0 N0 H1	No	Multiple	Normal	2	Nil
7	Female	1	Global DD	CMN PAS L2 > 30–40 cm CMN localization Extremities: upper leg Satellites S3 C0 R0 N0 H0	Yes	No	Intraparenchmyal melanosis	2	Infantile encephalopathicepilepsy Epithelioid melanocytoma on knee

MRI, magnetic resonance imaging; F, female; M, male; PAS, projected adult size; N/A, not applicable; DD, developmental delay.

Analysis of the association between *NRAS* mutant genotype (vs. *NRAS* wild‐type) and the PAS of the main CMN did not reveal a statistically significant association, with *NRAS* as the commonest mutation at all sizes of CMN, and the commonest in both single and multiple CMN. However, inspection of the raw data revealed that the percentage of patients with *NRAS‐*mutant CMN is fairly constant in all size groups (52–76%) except where PAS is > 60 cm, when 91% of patients (30 of 33) were *NRAS*‐mutant (Fig. 22a–c, Table 1).

**Figure 2:**
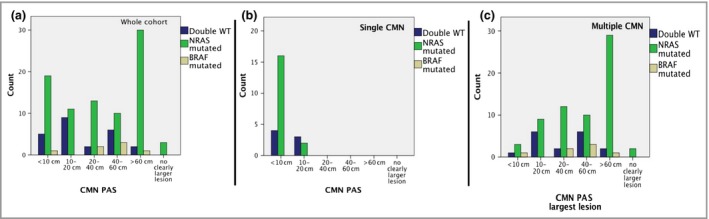
Interaction between congenital melanocytic naevus (CMN) projected adult size and genotype, for (a) the whole cohort (*n* = 134); (b) single naevus only; (c) multiple naevi only. While the *NRAS* mutant genotype is the commonest at all sizes of CMN, there is enrichment of this genotype specifically in the subgroup with projected adult size > 60cm. WT, wild‐type; PAS, projected adult size.

Typical facial features of CMN syndrome were significantly less common in double wild‐type patients compared with those carrying *NRAS* or *BRAF* mutations (*P* = 0·006). However, the numbers in the double wild‐type group with facial phenotyping were relatively small (*n* = 20), and this finding should therefore be interpreted as preliminary at this stage.

### Genotype–outcome analysis

**Figure 3:**
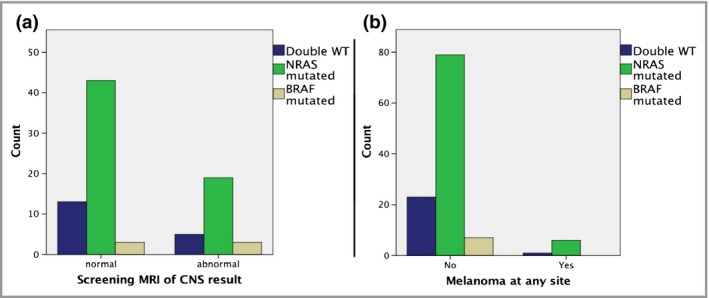
Interaction between genotype and (a) congenital neurological disease, and (b) melanoma incidence. No difference is observed between different groups. WT, wild‐type; MRI, magnetic resonance imaging; CNS, central nervous system.

No significant associations were found between genotype and presence of congenital neurological disease on screening MRI in the first 6–12 months of life (Fig. 3a). Cases of melanoma were described in the *NRAS*‐mutant and one in the double wild‐type group, and although no cases were described in *BRAF*‐mutant patients this is statistically compatible with the rarity of this genotype (Fig. 3b). Cases of melanoma were described across all *MC1R* genotyping groups (wild‐type, heterozygous, compound heterozygous/homozygous) (Table 1). Numbers of melanoma were too small to model this statistically.

### Histological characterization of *BRAF‐*mutant nodules

**Figure 4:**
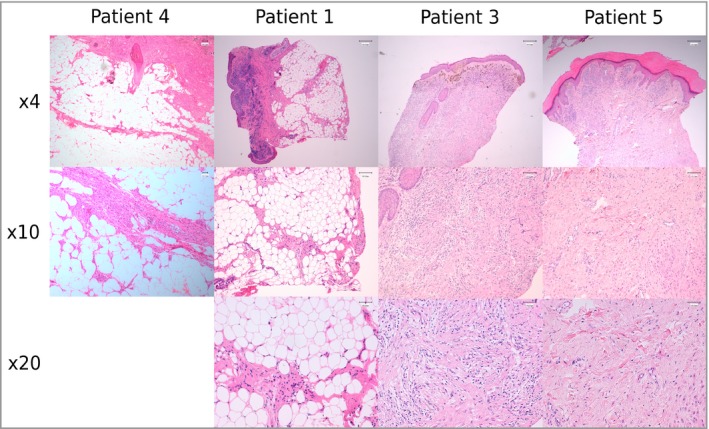
Histology of *BRAF* congenital melanocytic naevi (CMN) and nodules in four patients presenting with a multinodular CMN, showing proliferation of adipose tissue, with the naevus stretched across the top of the underlying proliferation, and a recurrent finding of septa‐like bands of naevus cells within the adipose tissue in patients 1, 3, 4 and 5 from Table 2.

Histopathological analysis of nodules demonstrated a pattern distinct from the classic proliferative nodules seen frequently in CMN, in which the proliferation is of naevus cells. In these *BRAF*‐mutant cases the proliferation instead appeared to be within the subcutis and adipose tissue, with the naevus itself stretched across the top of the underlying proliferation, and a recurrent finding of septa‐like bands of naevus cells within the adipose tissue (Fig. 4). This pattern has previously been described historically,^52^ but was not at that time connected to genotype.

### *NRAS/BRAF* mutations are heterozygous in naevus cells

**Figure 5:**
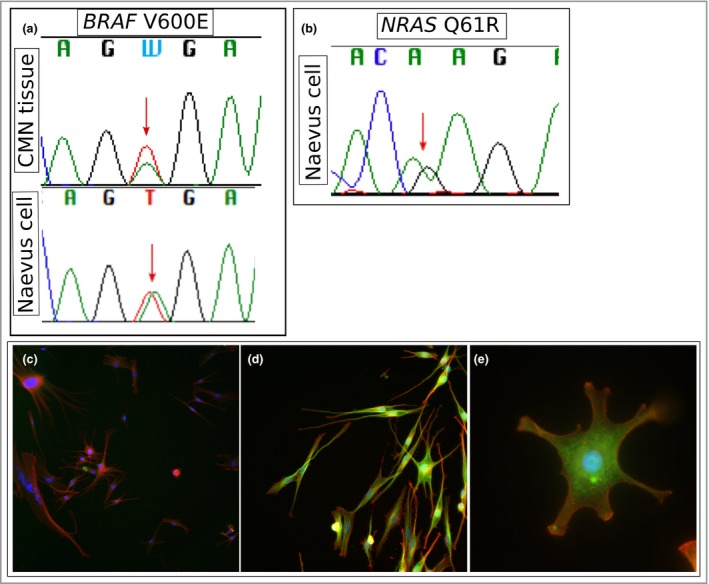
Sanger sequencing trace of DNA extracted directly from (a) congenital melanocytic naevus (CMN) tissue (upper) and DNA extracted from cultured naevus cells (lower) from the same patient, demonstrating *BRAF* c.1799T>A, p.(V600E) in both, which appears low‐level somatic in whole tissue and heterozygous in naevus cells. (b) A similar phenomenon is observed in naevus cells cultured directly from a patient with *NRAS* c183A>G, p.(Q61R). Immunocytochemistry of BRAF p.(V600E) naevus cells shows pancytoplasmic uniform expression of BRAF V600E (green) in all naevus cells. Cells are counterstained with Hoechst stain (blue) and Alexa Fluor 647‐conjugated phalloidin antibody (red). BRAF V600E negative control at original magnification × 20 (c), and BRAF V600E immunostaining at × 20 (d) and × 63 (e).

Sanger sequencing of DNA extracted directly from cultured naevus cells obtained from two patients, one with mutant *NRAS* p.(Q61R) and one with mutant *BRAF* p.(V600E), demonstrates heterozygosity for *NRAS/BRAF* mutations in naevus cells, with an increase in somatic mutation load in comparison with Sanger sequencing of DNA extracted directly from whole tissue from CMN (Fig. 5a, b), confirming that the naevus cells are the mutant cells.

### Naevus cells express mutant BRAF p.(V600E) in *BRAF* p.(V600E) CMN

Immunocytochemistry of primary naevus cells cultured from a *BRAF* p.(V600E) CMN demonstrates uniform expression of mutant BRAF protein in all naevus cells throughout the cytoplasm (Fig. 55d–f).

## Discussion

We confirm here in a large cohort of patients that *NRAS* mutations affecting codon 61 are the commonest cause of CMN, found in 68%, with *BRAF* mutations affecting codon 600 a much rarer cause at 7%. These percentages are comparable with the original description of 12 of 15 cases (80%) being *NRAS*,^37^ and with 77% and 8% *NRAS* and *BRAF*, respectively, described in another cohort of 66 patients with CMN.^40^ *BRAF* mutations as the first (or at least primary driver) hit as the cause of multiple CMN has only very recently been demonstrated,^44^ and has effectively confirmed previous reports of likely *BRAF* mosaicism in previous studies.^40^

*NRAS* and *BRAF* hotspot activating mutations have been found to be almost invariably mutually exclusive in melanoma and in other malignant tumours that commonly harbour these mutations.^53^, ^54^, ^55^, ^56^, ^57^, ^58^, ^59^, ^60^, ^61^ Rare incidences of concurrent *NRAS* and *BRAF* mutations have been described, for example in two cases of melanoma in a large series (0·7%),^62^ in one of 15 nodular melanomas,^63^ in one of 14 melanomas,^64^ in four of 60 cases of melanoma arising within an existing naevus,^65^ and in 1·6% in a large series of 484 melanomas; however in this last study, cloning of a single *NRAS/BRAF* mutant cell line revealed the mutations were mutually exclusive at single‐cell level.^64^ Although a number of studies have adopted sensitive techniques for *NRAS/BRAF* mutational analysis over Sanger sequencing for their improved ability to detect mutant alleles at low frequency,^34^, ^55^, ^64^, ^66^ many studies have relied on Sanger sequencing alone.^27^, ^30^, ^53^, ^54^, ^59^, ^67^, ^68^, ^69^ In our study, the use of at least two highly sensitive methods of mutation detection, and the unbiased genotyping strategy, can confirm mutual exclusivity of these mutations in CMN.

*BRAF* mutations appear to be commonly but not exclusively associated with a distinctive highly nodular phenotype, with characteristic clinical and histological appearances in five of seven cases. A previous cohort study demonstrated a significantly increased frequency of dermal/subcutaneous nodules in *BRAF‐* compared with *NRAS*‐mutated CMN,^40^ and the recent case of *BRAF* mosaicism also demonstrated a somewhat multinodular phenotype.^44^ NRAS is known to have multiple effector pathways, only one of which signals via BRAF, and it may be that the balance of these effectors is responsible for the differences in phenotype. Alternatively, constitutive BRAF activation via the p.(V600E) amino acid change may induce a different pattern of MAPK pathway overactivity. The apparent proliferation of subcutaneous adipose tissue as opposed to the naevus cells themselves suggests a non‐cell‐autonomous effect of the mutant naevus cells on the surrounding tissues. This finding is supported to some degree by a recent study of the histopathological features of acquired rather than congenital MN, which demonstrated differential morphology and cellular distribution in association with *BRAF* p.(V600E) expression.^70^

There is an interesting relationship between genotype and size of the main naevus, which has only become apparent in this large cohort. Previously it has been suggested that *BRAF* mutations are associated with smaller CMN, and *NRAS* with larger CMN.^30^, ^34^, ^66^ In one study, *BRAF* mutations were found in zero of 19 large CMN compared with six of 20 small/medium CMN^66^ and a statistically significant increased frequency of *BRAF* mutations in smaller sized CMN has been demonstrated in two papers, the first identifying *BRAF* mutations in 26 of 42 small CMN compared with only five of 20 medium CMN, and the second observing *BRAF* V600E in nine of 37 medium CMN compared with zero of 18 giant CMN.^34^ This apparent association was later called into question by a further paper describing *BRAF* V600E mutations in five of 66 CMN – in one of 20 patients with large CMN, and four of 36 patients with giant CMN.^40^ In our cohort, *NRAS* was clearly the commonest mutation in all PAS groups of CMN; however, a potential reason for the previous conflicting results is that in the group of > 60‐cm PAS there is a much higher frequency of *NRAS* mutations. As we know that these largest naevi are likely to be the first to form embryologically,^71^ the potential explanation for this is that *NRAS* mutations are more easily tolerated at that stage of embryogenesis than the non‐*NRAS* causes.

Neurological involvement has been described in two patients with *BRAF* p.(V600E) mutant ‘giant’ CMN.^40^ Data here from a large number of patients with MRI scans demonstrate a uniform pattern of congenital neurological abnormalities across the three genotypic groups. Similarly, data on melanoma in this cohort, although the numbers are small, do not suggest genotypic differences in outcome. No *BRAF*‐mutant patients had been diagnosed with melanoma, but this could be due to the small numbers rather than a genuinely differential genotypic effect. Going forward, the statistical significance of typical facies in patients appearing less commonly in those with double wild‐type genotype will need to be determined in a larger cohort. Potential limitations of this study are related to the numbers, as in particular the numbers of *BRAF* mutant patients are small. This will be resolved with a continuous prospective collection of this cohort.

We conclude here that *NRAS* mutations are the commonest cause of CMN, and of any size of CMN, being particularly common in the group with PAS > 60 cm. *BRAF* mutations can be a cause of multiple CMN, can be seen in any size group, and have a strong but not exclusive association with a distinct multinodular phenotype. Critically, clinical outcomes do not appear to be different among genotypic groups, with clear data on congenital neurological disease, and suggestive data for melanoma. Therefore, genotyping does not need to form part of routine clinical care. However, genotyping should be performed where melanoma is suspected, and particularly where melanoma treatment is required.

## References

[1] Castilla EE , da Graça Dutra M , Orioli‐Parreiras IM . Epidemiology of congenital pigmented naevi: I. Incidence rates and relative frequencies. Br J Dermatol 1981; 104:307–15. 7213564 10.1111/j.1365-2133.1981.tb00954.x

[2] Alper JC , Holmes LB . The incidence and significance of birthmarks in a cohort of 4,641 newborns. Pediatr Dermatol 1983; 1:58–68. 6679890 10.1111/j.1525-1470.1983.tb01093.x

[3] Jacobs AH , Walton RG . The incidence of birthmarks in the neonate. Pediatrics 1976; 58:218–22. 951136

[4] Wu PA , Mancini AJ , Marghoob AA , Frieden IJ . Simultaneous occurrence of infantile hemangioma and congenital melanocytic nevus: coincidence or real association? J Am Acad Dermatol 2008; 58 (Suppl. 1):S16–22. 18191691 10.1016/j.jaad.2006.04.085

[5] Kanada KN , Merin MR , Munden A , Friedlander SF . A prospective study of cutaneous findings in newborns in the United States: correlation with race, ethnicity, and gestational status using updated classification and nomenclature. J Pediatr 2012; 161:240–5. 22497908 10.1016/j.jpeds.2012.02.052

[6] Goss BD , Forman D , Ansell PE *et al* The prevalence and characteristics of congenital pigmented lesions in newborn babies in Oxford. Paediatr Perinat Epidemiol 1990; 4:448–57. 2267186 10.1111/j.1365-3016.1990.tb00672.x

[7] Shpall S , Frieden I , Chesney M , Newman T . Risk of malignant transformation of congenital melanocytic nevi in blacks. Pediatr Dermatol 1994; 11:204–8. 7971553 10.1111/j.1525-1470.1994.tb00587.x

[8] Krengel S , Hauschild A , Schafer T . Melanoma risk in congenital melanocytic naevi: a systematic review. Br J Dermatol 2006; 155:1–8. 10.1111/j.1365-2133.2006.07218.x 16792745

[9] Zaal LH , Mooi WJ , Klip H , van der Horst CM . Risk of malignant transformation of congenital melanocytic nevi: a retrospective nationwide study from the Netherlands. Plast Reconstr Surg 2005; 116:1902–9. 16327602 10.1097/01.prs.0000189205.85968.12

[10] Kinsler VA , O'Hare P , Bulstrode N *et al* Melanoma in congenital melanocytic naevi. Br J Dermatol 2017; 176:1131–43. 28078671 10.1111/bjd.15301 PMC5484991

[11] Kadonaga JN , Barkovich AJ , Edwards MS , Frieden IJ . Neurocutaneous melanosis in association with the Dandy‐Walker complex. Pediatr Dermatol 1992; 9:37–43. 1574474 10.1111/j.1525-1470.1992.tb00323.x

[12] Frieden IJ , Williams ML , Barkovich AJ . Giant congenital melanocytic nevi: brain magnetic resonance findings in neurologically asymptomatic children. J Am Acad Dermatol 1994; 31:423–9. 8077466 10.1016/s0190-9622(94)70204-7

[13] Waelchli R , Aylett SE , Atherton D *et al* Classification of neurological abnormalities in children with congenital melanocytic naevus syndrome identifies MRI as the best predictor of clinical outcome. Br J Dermatol 2015; 173:739–50. 25966033 10.1111/bjd.13898 PMC4737261

[14] Ramaswamy V , Delaney H , Haque S *et al* Spectrum of central nervous system abnormalities in neurocutaneous melanocytosis. Dev Med Child Neurol 2012; 54:563–8. 22469364 10.1111/j.1469-8749.2012.04275.x

[15] Kinsler V , Shaw AC , Merks JH , Hennekam RC . The face in congenital melanocytic nevus syndrome. Am J Med Genet A 2012; 158a:1014–19. 22438093 10.1002/ajmg.a.34217

[16] Waelchli R , Williams J , Cole T *et al* Growth and hormone profiling in children with congenital melanocytic naevi. Br J Dermatol 2015; 173:1471–8. 26286459 10.1111/bjd.14091 PMC4737097

[17] Kinsler VA , O'Hare P , Bulstrode N *et al* Melanoma in congenital melanocytic naevi. Br J Dermatol 2017; 176:1131–43. 28078671 10.1111/bjd.15301 PMC5484991

[18] Cantu JM , Urrusti J , Hernandez A *et al* Discordance for giant pigmented nevi in monozygotic twins. Ann Genet 1973; 16:289–92. 4544096

[19] Amir J , Metzker A , Nitzan M . Giant pigmented nevus occurring in one identical twin. Arch Dermatol 1982; 118:188–9. 7199895

[20] Happle R . Lethal genes surviving by mosaicism: a possible explanation for sporadic birth defects involving the skin. J Am Acad Dermatol 1987; 16:899–906. 3033033 10.1016/s0190-9622(87)80249-9

[21] de Wijn RS , Zaal LH , Hennekam RC , van der Horst CM . Familial clustering of giant congenital melanocytic nevi. J Plast Reconstr Aesthet Surg 2010; 63:906–13. 19464972 10.1016/j.bjps.2009.02.090

[22] Rhodes AR , Slifman NR , Korf BR . Familial aggregation of small congenital nevomelanocytic nevi. Am J Med Genet 1985; 22:315–26. 4050864 10.1002/ajmg.1320220215

[23] Frieden IJ , Williams ML . Familial site‐specific congenital melanocytic nevus: report of two families. Arch Dermatol 1994; 130:1075–6. 8053712

[24] Danarti R , König A , Happle R . Large congenital melanocytic nevi may reflect paradominant inheritance implying allelic loss. Eur J Dermatol 2003; 13:430–2. 14693484

[25] Happle R . Giant melanocytic nevus may be explained as a superimposed patchy manifestation of a polygenic trait. Dermatology 2010; 221:30–3. 20424416 10.1159/000278229

[26] Papp T , Pemsel H , Zimmermann R *et al* Mutational analysis of the *N‐ras*,* p53*,* p16* ^*INK4a*^, *CDK4*, and *MC1R* genes in human congenital melanocytic naevi. J Med Genet 1999; 36:610–14. 10465111 PMC1762982

[27] Bauer J , Curtin JA , Pinkel D , Bastian BC . Congenital melanocytic nevi frequently harbor *NRAS* mutations but no *BRAF* mutations. J Invest Dermatol 2007; 127:179–82. 16888631 10.1038/sj.jid.5700490

[28] Dessars B , De Raeve LE , Morandini R *et al* Genotypic and gene expression studies in congenital melanocytic nevi: insight into initial steps of melanotumorigenesis. J Invest Dermatol 2009; 129:139–47. 18633438 10.1038/jid.2008.203

[29] Phadke PA , Rakheja D , Le LP *et al* Proliferative nodules arising within congenital melanocytic nevi: a histologic, immunohistochemical, and molecular analyses of 43 cases. Am J Surg Pathol 2011; 35:656–69. 21436676 10.1097/PAS.0b013e31821375ea

[30] Wu D , Wang M , Wang X *et al* Lack of *BRAFV600E* mutations in giant congenital melanocytic nevi in a Chinese population. Am J Dermatopathol 2011; 33:341–4. 21430505 10.1097/DAD.0b013e3181fb5bc7

[31] Pollock PM , Harper UL , Hansen KS *et al* High frequency of *BRAF* mutations in nevi. Nat Genet 2003; 33:19–20. 12447372 10.1038/ng1054

[32] Kumar R , Angelini S , Snellman E , Hemminki K . *BRAF* mutations are common somatic events in melanocytic nevi. J Invest Dermatol 2004; 122:342–8. 15009715 10.1046/j.0022-202X.2004.22225.x

[33] Papp T , Schipper H , Kumar K *et al* Mutational analysis of the *BRAF* gene in human congenital and dysplastic melanocytic naevi. Melanoma Res 2005; 15:401–7. 16179867 10.1097/00008390-200510000-00008

[34] Ichii‐Nakato N , Takata M , Takayanagi S *et al* High frequency of *BRAF* ^*V600E*^ mutation in acquired nevi and small congenital nevi, but low frequency of mutation in medium‐sized congenital nevi. J Invest Dermatol 2006; 126:2111–18. 16691193 10.1038/sj.jid.5700366

[35] Dessars B , De Raeve LE , El Housni H *et al* Chromosomal translocations as a mechanism of *BRAF* activation in two cases of large congenital melanocytic nevi. J Invest Dermatol 2007; 127:1468–70. 17301836 10.1038/sj.jid.5700725

[36] Kinsler VA , Abu‐Amero S , Budd P *et al* Germline melanocortin‐1‐receptor genotype is associated with severity of cutaneous phenotype in congenital melanocytic nevi: a role for MC1R in human fetal development. J Invest Dermatol 2012; 132:2026–32. 22572819 10.1038/jid.2012.95 PMC3398254

[37] Kinsler VA , Thomas AC , Ishida M *et al* Multiple congenital melanocytic nevi and neurocutaneous melanosis are caused by postzygotic mutations in codon 61 of *NRAS* . J Invest Dermatol 2013; 133:2229–36. 23392294 10.1038/jid.2013.70 PMC3678977

[38] Kinsler VA , Krengel S , Riviere JB *et al* Next‐generation sequencing of nevus spilus‐type congenital melanocytic nevus: exquisite genotype–phenotype correlation in mosaic RASopathies. J Invest Dermatol 2014; 134:2658–60. 24751729 10.1038/jid.2014.195 PMC4165863

[39] Krengel S , Widmer DS , Kerl K *et al* Naevus spilus‐type congenital melanocytic naevus associated with a novel *NRAS* codon 61 mutation. Br J Dermatol 2015; 174:642–4. 26302237 10.1111/bjd.14105

[40] Salgado CM , Basu D , Nikiforova M *et al* *BRAF* mutations are also associated with neurocutaneous melanocytosis and large/giant congenital melanocytic nevi. Pediatr Dev Pathol 2015; 18:1–9. 25490715 10.2350/14-10-1566-OA.1

[41] Charbel C , Fontaine RH , Malouf GG *et al* *NRAS* mutation is the sole recurrent somatic mutation in large congenital melanocytic nevi. J Invest Dermatol 2014; 134:1067–74. 24129063 10.1038/jid.2013.429

[42] Mehraein Y , Ehlhardt S , Wagner A *et al* Somatic mosaicism of chromosome 7 in a highly proliferating melanocytic congenital naevus in a ring chromosome 7 patient. Am J Med Genet A 2004; 131:179–85. 15523614 10.1002/ajmg.a.30370

[43] Salas‐Labadía C , Cervantes‐Barragán DE , Cruz‐Alcívar R *et al* Cytogenomic and phenotypic analysis in low‐level monosomy 7 mosaicism with non‐supernumerary ring chromosome 7. Am J Med Genet A 2014; 164:1765–9. 10.1002/ajmg.a.36503 24677512

[44] Etchevers HC , Rose C , Kahle B *et al* Giant congenital melanocytic nevus with vascular malformation and epidermal cysts associated with a somatic activating mutation in *BRAF* . Pigment Cell Melanoma Res 2018; 31:437–41. 29316280 10.1111/pcmr.12685

[45] Martins da Silva V , Martinez‐Barrios E , Tell‐Martí G *et al* Genetic abnormalities in large to giant congenital nevi: beyond *NRAS* mutations. J Invest Dermatol 2018; 139:900–9. 30359577 10.1016/j.jid.2018.07.045

[46] Kinsler VA , O'Hare P , Jacques T *et al* MEK inhibition appears to improve symptom control in primary *NRAS*‐driven CNS melanoma in children. Br J Cancer 2017; 116:990–3. 28253523 10.1038/bjc.2017.49 PMC5396107

[47] Krengel S , Scope A , Dusza SW *et al* New recommendations for the categorization of cutaneous features of congenital melanocytic nevi. J Am Acad Dermatol 2013; 68:441–51. 22982004 10.1016/j.jaad.2012.05.043

[48] Forbes SA , Beare D , Gunasekaran P *et al* COSMIC: exploring the world's knowledge of somatic mutations in human cancer. Nucleic Acids Res 2015; 43:D805–11. 25355519 10.1093/nar/gku1075 PMC4383913

[49] Cancer Genome Atlas Network . Genomic Classification of Cutaneous Melanoma. Cell 2015; 161:1681–96. 26091043 10.1016/j.cell.2015.05.044 PMC4580370

[50] Gallagher SJ , Thompson JF , Indsto J *et al* p16^INK4a^ expression and absence of activated *B‐RAF* are independent predictors of chemosensitivity in melanoma tumors. Neoplasia 2008; 10:1231–9. 18953432 10.1593/neo.08702 PMC2570599

[51] Ellerhorst JA , Greene VR , Ekmekcioglu S *et al* Clinical correlates of *NRAS* and *BRAF* mutations in primary human melanoma. Clin Cancer Res 2011; 17:229–35. 20975100 10.1158/1078-0432.CCR-10-2276 PMC3022950

[52] Reed RJ . Giant congenital nevi: a conceptualization of patterns. J Invest Dermatol 1993; 100:300s–12s. 8440910 10.1111/1523-1747.ep12470191

[53] Brose MS , Volpe P , Feldman M *et al* *BRAF* and *RAS* mutations in human lung cancer and melanoma. Cancer Res 2002; 62:6997–7000. 12460918

[54] Daniotti M , Oggionni M , Ranzani T *et al* BRAF alterations are associated with complex mutational profiles in malignant melanoma. Oncogene 2004; 23:5968–77. 15195137 10.1038/sj.onc.1207780

[55] Goydos JS , Mann B , Kim HJ *et al* Detection of *B‐RAF* and *N‐RAS* mutations in human melanoma. J Am Coll Surg 2005; 200:362–70. 15737846 10.1016/j.jamcollsurg.2004.10.032

[56] Hale EK , Stein J , Ben‐Porat L *et al* Association of melanoma and neurocutaneous melanocytosis with large congenital melanocytic naevi – results from the NYU–LCMN registry. Br J Dermatol 2005; 152:512–17. 15787820 10.1111/j.1365-2133.2005.06316.x

[57] Platz A , Egyhazi S , Ringborg U , Hansson J . Human cutaneous melanoma; a review of *NRAS* and *BRAF* mutation frequencies in relation to histogenetic subclass and body site. Mol Oncol 2008; 1:395–405. 19383313 10.1016/j.molonc.2007.12.003 PMC5543839

[58] Tschandl P , Berghoff AS , Preusser M *et al* NRAS and BRAF mutations in melanoma‐associated nevi and uninvolved nevi. PLOS ONE 2013; 8:e69639. 23861977 10.1371/journal.pone.0069639 PMC3704624

[59] Zebary A , Jangard M , Omholt K *et al* *KIT*,* NRAS* and *BRAF* mutations in sinonasal mucosal melanoma: a study of 56 cases. Br J Cancer 2013; 109:559–64. 23860532 10.1038/bjc.2013.373 PMC3738146

[60] Kunstman JW , Juhlin CC , Goh G *et al* Characterization of the mutational landscape of anaplastic thyroid cancer via whole‐exome sequencing. Hum Mol Genet 2015; 24:2318–29. 25576899 10.1093/hmg/ddu749 PMC4380073

[61] Lu C , Zhang J , Nagahawatte P *et al* The Genomic Landscape of Childhood and Adolescent Melanoma. J Invest Dermatol 2015; 135:816–23. 25268584 10.1038/jid.2014.425 PMC4340976

[62] Edlundh‐Rose E , Egyházi S , Omholt K *et al* *NRAS* and *BRAF* mutations in melanoma tumours in relation to clinical characteristics: a study based on mutation screening by pyrosequencing. Melanoma Res 2006; 16:471–8. 17119447 10.1097/01.cmr.0000232300.22032.86

[63] Chiappetta C , Proietti I , Soccodato V *et al* BRAF and NRAS mutations are heterogeneous and not mutually exclusive in nodular melanoma. Appl Immunohistochem Mol Morphol 2015; 23:172–7. 24710085 10.1097/PAI.0000000000000071 PMC4482453

[64] Sensi M , Nicolini G , Petti C *et al* Mutually exclusive *NRAS* ^*Q61R*^ and *BRAF* ^*V600E*^ mutations at the single‐cell level in the same human melanoma. Oncogene 2006; 25:3357–64. 16462768 10.1038/sj.onc.1209379

[65] Shitara D , Tell‐Martí G , Badenas C *et al* Mutational status of naevus associated‐melanomas. Br J Dermatol 2015; 173:671–80. 25857817 10.1111/bjd.13829 PMC4583836

[66] Charbel C , Fontaine RH , Malouf GG *et al* *NRAS* mutation is the sole recurrent somatic mutation in large congenital melanocytic nevi. J Invest Dermatol 2014; 134:1067–74. 24129063 10.1038/jid.2013.429

[67] Poynter JN , Elder JT , Fullen DR *et al* BRAF and NRAS mutations in melanoma and melanocytic nevi. Melanoma Res 2006; 16:267–73. 16845322 10.1097/01.cmr.0000222600.73179.f3

[68] Si L , Kong Y , Xu X *et al* Prevalence of BRAF V600E mutation in Chinese melanoma patients: large scale analysis of BRAF and NRAS mutations in a 432‐case cohort. Eur J Cancer 2012; 48:94–100. 21788131 10.1016/j.ejca.2011.06.056

[69] Colombino M , Lissia A , Capone M *et al* Heterogeneous distribution of *BRAF/NRAS* mutations among Italian patients with advanced melanoma. J Transl Med 2013; 11:202. 23987572 10.1186/1479-5876-11-202 PMC3765741

[70] Kiuru M , Tartar DM , Qi L *et al* Improving classification of melanocytic nevi: BRAF V600E expression associated with distinct histomorphologic features. J Am Acad Dermatol 2018; 79:221–9. 29653212 10.1016/j.jaad.2018.03.052 PMC6090558

[71] Kinsler VA , Larue L . The patterns of birthmarks suggest a novel population of melanocyte precursors arising around the time of gastrulation. Pigment Cell Melanoma Res 2018; 31:95–109. 28940934 10.1111/pcmr.12645 PMC5765478

